# Validation of an optimized Oxford Nanopore sequencing workflow versus Illumina for mycobacteria from primary MGIT culture

**DOI:** 10.1128/spectrum.01402-26

**Published:** 2026-08-20

**Authors:** Catriona S. Baker, Matthew Colpus, Jess Gentry, Alexandra Hall, Eloïse Roghi, Hermione Webster, Bronte Drummond, Ruth Cooper, Hieu Thai, Jeremy Westhead, Robert Turner, Timothy E. A. Peto, Philip W. Fowler, Marcus Morgan, Derrick W. Crook

**Affiliations:** 1 Modernising Medical Microbiology Unit, Nuffield Department of Medicine, University of Oxford 6396, Oxford, United Kingdom; 2 Oxford University Hospitals NHS Foundation Trust 6397 , Oxford, England, United Kingdom; 3 National Institute of Health Research Oxford Biomedical Research Center, University of Oxford 6396, Oxford, United Kingdom; 4 Health Protection Research Unit in Healthcare Associated Infections and Antimicrobial Resistance, John Radcliffe Hospital 11269, Oxford, United Kingdom; 5 Shared Hospital Laboratory and Sunnybrook Research Institute, Sunnybrook Hospital 71545, Toronto, Ontario, Canada; Cincinnati Children's Hospital Medical Center, Cincinnati, Ohio, USA

**Keywords:** mycobacteria, whole-genome sequencing, DNA extraction, long-read sequencing

## Abstract

Illumina short-read sequencing of primary Mycobacterial Growth Indicator Tube (MGIT) cultures is an established approach for mycobacterial genomic characterization but relies on labor-intensive workflows and centralized sequencing facilities. Oxford Nanopore Technologies (ONT) offers long-read sequencing and is increasingly being explored for in-house implementation in diagnostic laboratories; however, low DNA yields from primary MGIT cultures frequently limit standard PCR-free ONT workflows, restricting routine implementation. We developed and evaluated a semi-automated DNA extraction and Rapid PCR Barcoding workflow for ONT sequencing and compared its performance with Illumina for species identification and *Mycobacterium tuberculosis* complex (MTBC) single-nucleotide polymorphism (SNP) detection. A platform-agnostic bioinformatics pipeline was used for human read removal, taxonomic assignment, and MTBC genomic characterization. The time required to achieve reliable species identification was assessed by subsampling ONT data at 1, 6, and 72 h. Concordance between ONT and Illumina for species classification was 98.3% (95% CI: 95.8%–99.5%), with all discordant classifications attributable to potential mixed infections. MTBC SNP concordance was high, with a mean of 0.3 and a median of 0 SNP differences between platforms after masking. Reliable species identification was achieved for 233/237 (98.3%) samples within 6 h of sequencing, with no incorrect primary species assignments. These findings demonstrate that an optimized PCR-amplified ONT workflow provides a practical approach for routine in-house genomic characterization of primary MGIT cultures, achieving high concordance with Illumina while addressing a major barrier to ONT implementation in diagnostic laboratories.

**IMPORTANCE:**

Rapid genomic characterization of mycobacterial infections is important for timely patient management, infection control, and public health surveillance. However, many diagnostic laboratories rely on referral of positive cultures to regional reference centers for sequencing, introducing delays associated with transport, batching, and processing. Although Oxford Nanopore Technologies (ONT) can support local sequencing, implementation has been limited by the low DNA yields typically obtained from primary Mycobacterial Growth Indicator Tube (MGIT) cultures and the absence of practical workflows suitable for routine diagnostic laboratories. In this study, we developed and validated a semi-automated DNA extraction and PCR-based ONT workflow designed to support implementation in routine diagnostic laboratories. The workflow generated genomic results highly concordant with Illumina sequencing while overcoming a major barrier to ONT implementation by enabling reliable sequencing from low-yield MGIT-derived DNA. This approach supports routine in-house genomic characterization of mycobacterial cultures and has the potential to reduce turnaround times and dependence on external sequencing services.

## INTRODUCTION

The genus *Mycobacterium* comprises more than 190 recognized species, many of which are capable of causing a wide range of human diseases, including pulmonary, cutaneous, and systemic infections (1, 2). Globally, the *Mycobacterium tuberculosis* complex (MTBC) remains a leading cause of infectious disease, with an estimated 10.7 million new cases and 1.23 million deaths worldwide due to tuberculosis (TB) in 2024 (3). In addition, non-tuberculous mycobacteria (NTM) are emerging in prominence as opportunistic pathogens, particularly among older individuals and those with impaired host defenses, with associated mortality more than doubling in the last two decades (4).

Although traditional culture-based identification of *Mycobacterium* species is often slow, species identification has improved considerably with the adoption of modern platforms such as matrix-assisted laser desorption/ionization-time of flight mass spectrometry, which rapidly and accurately identifies many NTM species (5). However, for *Mycobacterium* spp., particularly the MTBC, whole-genome sequencing (WGS) provides substantially greater resolution, enabling precise species differentiation, lineage assignment, antimicrobial-resistance (AMR) prediction, and high-resolution single-nucleotide polymorphism (SNP)-based epidemiological analyses from a single assay (6).

In the UK, WGS of mycobacterial isolates is typically performed in regional reference laboratories, resulting in delays associated with sample transport, batching, and sequencing. Wider implementation of WGS within hospital diagnostic laboratories has also been constrained by upstream sample preparation. Conventional DNA extraction methods, such as cetyltrimethylammonium bromide, are labor-intensive, time-consuming, and poorly suited to automation or high-throughput processing. Together, these limitations have hindered the adoption of rapid, in-house sequencing workflows despite advances in sequencing technology. Reducing dependence on regional reference laboratories therefore requires workflows that are rapid, robust, and compatible with routine diagnostic practice.

Illumina sequencing remains the most widely adopted approach due to its maturity and high base-calling accuracy and is often regarded as the gold standard when assessing newer technologies (7). This accuracy achieved by Illumina platforms confers a major advantage for reliable SNP detection (8). However, Illumina sequencing typically generates reads of up to 300 bp, which complicates the resolution of repetitive or complex genomic regions (e.g., the PE/PPE gene family in *M. tuberculosis*), potentially leading to incomplete genomic assemblies (9, 10).

Long-read sequencing with Oxford Nanopore Technologies (ONT) offers several technical advantages, including improved resolution of repetitive genomic regions and rapid data generation. Recent improvements in ONT chemistry and hardware, particularly the R10.4.1 flow cells with V14 chemistry, have improved base-calling accuracy, bringing ONT performance closer to that of Illumina (11, 12).

Nonetheless, routine implementation of ONT sequencing for primary Mycobacterial Growth Indicator Tube (MGIT) cultures remains challenging because DNA yields frequently fall below the input requirements of current library preparation kits. For ONT sequencing, insufficient input DNA frequently results in low coverage and depth, limiting species identification and preventing reliable AMR prediction (7, 13, 14). These constraints necessitate the use of PCR-based ONT library preparation strategies when working with low-yield MGIT-derived DNA.

A streamlined workflow for sequencing directly from positive MGIT using ONT has not previously been described, limiting the routine implementation of ONT in clinical mycobacteriology. Here, we describe a semi-automated DNA extraction method and modified Rapid PCR Barcoding workflow optimized for low-yield MGIT-derived DNA and evaluate its performance against Illumina sequencing for *Mycobacterium* species identification, MTBC SNP detection, and antimicrobial resistance prediction. In addition, we assessed the earliest reliable time point for species identification using the optimized ONT workflow. Our aim was to develop and validate a practical workflow suitable for routine in-house genomic characterization of positive MGIT cultures within diagnostic microbiology laboratories.

## MATERIALS AND METHODS

### Sample processing

Samples submitted to the Oxford University Hospitals (OUHs) microbiology laboratory for detection of *Mycobacterium* spp. between September 2024 and September 2025 were processed according to specimen type. Non-sterile specimens were decontaminated with sodium hydroxide, whereas sterile specimens were cultured without prior decontamination. Following processing, specimens were inoculated into MGIT (BD) supplemented with PANTA (polymyxin B, amphotericin B, nalidixic acid, trimethoprim, and azlocillin) and incubated at 37°C for up to 56 days. Mycobacterial growth was detected using the BD BACTEC MGIT system, and culture positivity was confirmed by Ziehl–Neelsen staining for acid-fast bacilli (AFB).

An aliquot of 1 mL from each positive MGIT tube was stored at 2°C–8°C for up to 5 days prior to batching for further processing. Samples were then incubated at 37°C for 5 days to enhance growth, centrifuged at 6,000 *g* for 5 min, and the resulting pellet was resuspended in 200 μL phosphate-buffered saline (EO Labs) in a 1.5 mL Sarstedt screw-top tube.

Each sample was analyzed separately, and multiple samples from the same patient were included where available (i.e., patient samples were not de-duplicated). Duplicate samples collected within different time frames were available for three patients.

### Heat inactivation and validation

Samples were heat inactivated in a fan-assisted oven at 80°C for 30 min. After heat inactivation, samples were transferred to a Containment Level two laboratory. Heat inactivation of *Mycobacterium* spp. was validated by OUH Microbiology, using 20 *Mycobacterium* isolates in MGIT fluid processed as described above, with no growth detected following 56 days of incubation.

### Cell lysis

The extraction workflow is adapted from George et al. (15). Lysing Matrix B beads (0.08–0.10 g; MP Biomedicals, CA, USA) were added to 200 μL heat-inactivated sample before undergoing two rounds of mechanical lysis using the MP Bio FastPrep 24 (MP Biomedicals, CA, USA) at 6.0 m/s for 40 s with a 5-min interval between lysis rounds. Lysed supernatant was collected following centrifugation at 16,000*g* for 10 min at room temperature.

### DNA purification

Lysed supernatant underwent DNA purification using the KingFisher Flex (Thermo Scientific) system with the addition of 1× AMPure XP beads (Beckman Coulter, CA, USA) and washed twice with 70% ethanol and then eluted into 50 μL elution buffer (CDT-01, Quickgene).

### Quantification

Extracted DNA was measured using a Qubit 4.0 fluorometer with the 1× High Sensitivity dsDNA kit, or for samples >120 ng/μL, the 1× Broad Range dsDNA kit (Invitrogen, CA, USA).

Illumina sequencing was commissioned from a commercial supplier, GENEWIZ Germany GmbH (Leipzig, Germany), which recommends a minimum DNA concentration of 6 ng/μL for its Whole-Genome Sequencing (human/non-human) library preparation workflow. For ONT sequencing, the Rapid PCR Barcoding workflow requires a minimum DNA input of 1 ng in 3 μL (equivalent to around 0.334 ng/μL). To enable paired ONT–Illumina comparative analyses while maximizing sample inclusion, an internal DNA concentration threshold of 1 ng/μL was applied, and samples below this threshold were excluded from downstream analyses. In addition, six samples with DNA concentrations below this threshold were sequenced using the ONT workflow alone to assess the feasibility of low-input sequencing (Table S2).

#### Comparison with the previous extraction method

To compare DNA yield with a previously used extraction protocol, 154 MGIT-positive samples were processed using an earlier adaptation of the method described by George et al. (15). Briefly, MGIT cultures were heat inactivated in 1 mL Thermo Protection Buffer at 99°C for 30 min before undergoing cell lysis, DNA purification, and quantification as described above. Samples were processed only to the DNA quantification stage and were not sequenced.

### Sequencing

#### Illumina

Short-read sequencing was performed by a commercial provider as described above. Library preparation was performed using the NEBNext Ultra II DNA Library Preparation Kit, and Illumina clustering and sequencing were carried out in accordance with the manufacturer’s instructions.

Briefly, genomic DNA was fragmented by acoustic shearing using a Covaris instrument, followed by end repair and 3′-end adenylation. Adapters were ligated, followed by enrichment by limited-cycle PCR. Libraries were validated using the Agilent 5600 Fragment Analyzer and quantified using a Qubit 4.0 Fluorometer.

Validated libraries were multiplexed and loaded onto an Illumina NovaSeq X Plus instrument according to the manufacturer’s instructions. Sequencing was performed using a 2 × 150 bp paired-end configuration. Image analysis and base calling were performed using NovaSeq Control Software. Raw sequencing data (.bcl files) were converted to FASTQ files and demultiplexed using Illumina bcl2fastq software.

#### Oxford Nanopore Technologies

ONT sequencing was performed using the Rapid PCR Barcoding Kit (SQK-RPB114.24) rather than a PCR-free protocol, in accordance with the manufacturer’s recommendations for low-input DNA samples. Sequencing was performed on R10.4.1 flow cells using v14 chemistry. Libraries were multiplexed with 22–24 samples across 10 flow cells; 2 additional flow cells were multiplexed with 8 and 11 samples per library. Only flow cells with more than 800 active pores (within warranty) were used.

The workflow was optimized by substituting Ex Premier DNA Polymerase (TaKaRa) for the recommended LongAmp Hot Start Taq 2× Master Mix. This substitution was selected following optimization experiments using Bacillus Calmette-Guérin (BCG)-spiked bronchoalveolar lavage samples, in which Ex Premier provided more consistent PCR amplification and higher DNA yields (Table S1). PCR cycling conditions were subsequently optimized for this study and are shown in Table 1.

**TABLE 1:** PCR cycling conditions used in the modified Rapid PCR Barcoding workflow including initial denaturation, 30 amplification cycles, and final extension

Step	Temperature (°C)	Time (min:s)	
Initial	94	1:00	
Denature	98	0:10	
Anneal	56	0:15	30 cycles
Extend	68	6:00	
Final extension	65	6:00	
	4	Hold	

Apart from these modifications, the manufacturer’s protocol for the SQK-RPB114.24 kit was followed. SUP basecalling was used with Dorado 4.3.0 through the onboard MinKNOW software on an ONT GridION.

#### Controls

For both ONT and Illumina sequencing, *Mycobacterium tuberculosis* H37Rv was included as a positive control in sequencing runs (Illumina *n* = 4, ONT *n* = 2). One *Mycobacterium bovis* BCG Pasteur strain sample was additionally sequenced using ONT. H37Rv and BCG isolates were obtained from laboratory reference stocks.

### Bioinformatics

Sequencing data were processed using an instance of an online cloud platform (16). All samples had human reads removed using Hostile (v1.1.0) or Deacon (v0.7.0) (17, 18), before quality filtering with Fastp (v0.24.1) (19). Kraken2 (v2.1.3) was used to select reads from the *Mycobacteriaceae* family (using standard index 6 May 2023) (20). Sylph (v0.9.0) was used after Kraken2 for species identification using the GTDB taxonomy (21). Reads were mapped using Minimap2 (v2.28 with “map-ont” preset) against a multifasta of *Mycobacterium* reference genomes to select for MTBC reads and to provide an alternative method of mycobacterial species identification (competitive mapping), while Mykrobe (v0.13.0) was used to determine lineage (22, 23).

MTBC reads were used for variant calling against the H37Rv reference using Clockwork (v0.12.4) for Illumina, and Clair3 (v1.1.2)/BCFTools (v1.22) for ONT (24–27). Variants required a minimum read support of two reads for Illumina and five reads for ONT. Gnomonicus (v3.1.1) was used to predict resistance according to the second edition of the WHO catalog (WHOv2) (16, 28). Uncatalogued mutations in resistance-associated genes were classified as “Unknown,” while a result of “Fail” was returned when there were insufficient reads at a genetic locus known to be associated with resistance.

Pairwise relatedness was assessed using masked SNP distances (12). The mask excludes repetitive and low complexity regions, as well as regions with frequent errors when mapping Illumina reads (29). SNPs within 12 base-pairs of another SNP were also excluded (30).

The Clopper-Pearson exact binomial method was used to calculate 95% confidence intervals.

#### Time to reliable species identification

To determine the earliest reliable time point for species identification, ONT sequencing data were subsampled at 1, 6, and 72 h. MinKNOW was configured to generate per-barcode POD5 files at hourly intervals throughout each sequencing run. Following completion of the run, POD5 files were basecalled and combined to generate FASTQ data sets corresponding to each time point. Each data set was analyzed independently using the same bioinformatic pipeline as the complete 72-h data set, detailed above.

## RESULTS

### Performance of the optimized extraction workflow

The optimized extraction workflow generated sufficient DNA to support ONT sequencing from the majority of primary MGIT cultures across a range of specimen types (Table 2). DNA extracted from 264 clinical MGIT cultures had a median concentration of 12.4 ng/μL. A previous evaluation of an earlier adaptation of the George et al. workflow (15) yielded a median DNA concentration of 0.5 ng/μL from 154 MGIT cultures.

**TABLE 2:** Specimen types of the 264 MGIT cultures included in the study^*a*^

Specimen type	Number
Sputum	206 (78%)
Bronchoalveolar lavage	26 (10%)
Pus	5 (2%)
Other	27 (10%)

^*a*^ Other includes pleural fluid, bone biopsy, soft tissue, cerebrospinal fluid, and other less frequently encountered specimen types.

The ONT Rapid Barcoding Kit recommends a total DNA input of 200 ng in 10 µL (equivalent to approximately 20 ng/μL) for PCR-free library preparation. Based on this recommendation, 66% (175/264) of samples remained below the manufacturer’s recommended input threshold, necessitating PCR-based library preparation.

An internal threshold of 1 ng/µL resulted in 250/264 samples being included in the paired ONT–Illumina comparison. Fourteen samples were excluded because insufficient DNA remained to perform sequencing on both platforms from the same extract.

To investigate whether sequencing remained feasible below this threshold, six additional MGIT cultures with DNA concentrations ranging from 0.330 to 0.63 ng/μL (median 0.47 μL) were processed using the ONT workflow alone. Following PCR amplification with Ex Premier, DNA concentrations increased to 31.9–86.6 ng/μL (median 66.5 ng/μL). All six generated sufficient sequencing data for species identification (Table S2), including three MTBC and three NTM isolates. MTBC lineage assignment was successful for all three TB samples. These samples were not included in the main comparative analyses because matched Illumina sequencing data were unavailable.

All 250 samples meeting the internal study threshold were successfully sequenced using Illumina. Five samples failed PCR amplification and were therefore excluded from ONT sequencing. Among the remaining 245 samples, PCR amplification increased the median DNA concentration from 13 ng/μL to 36.6 ng/μL.

**Fig 1:**
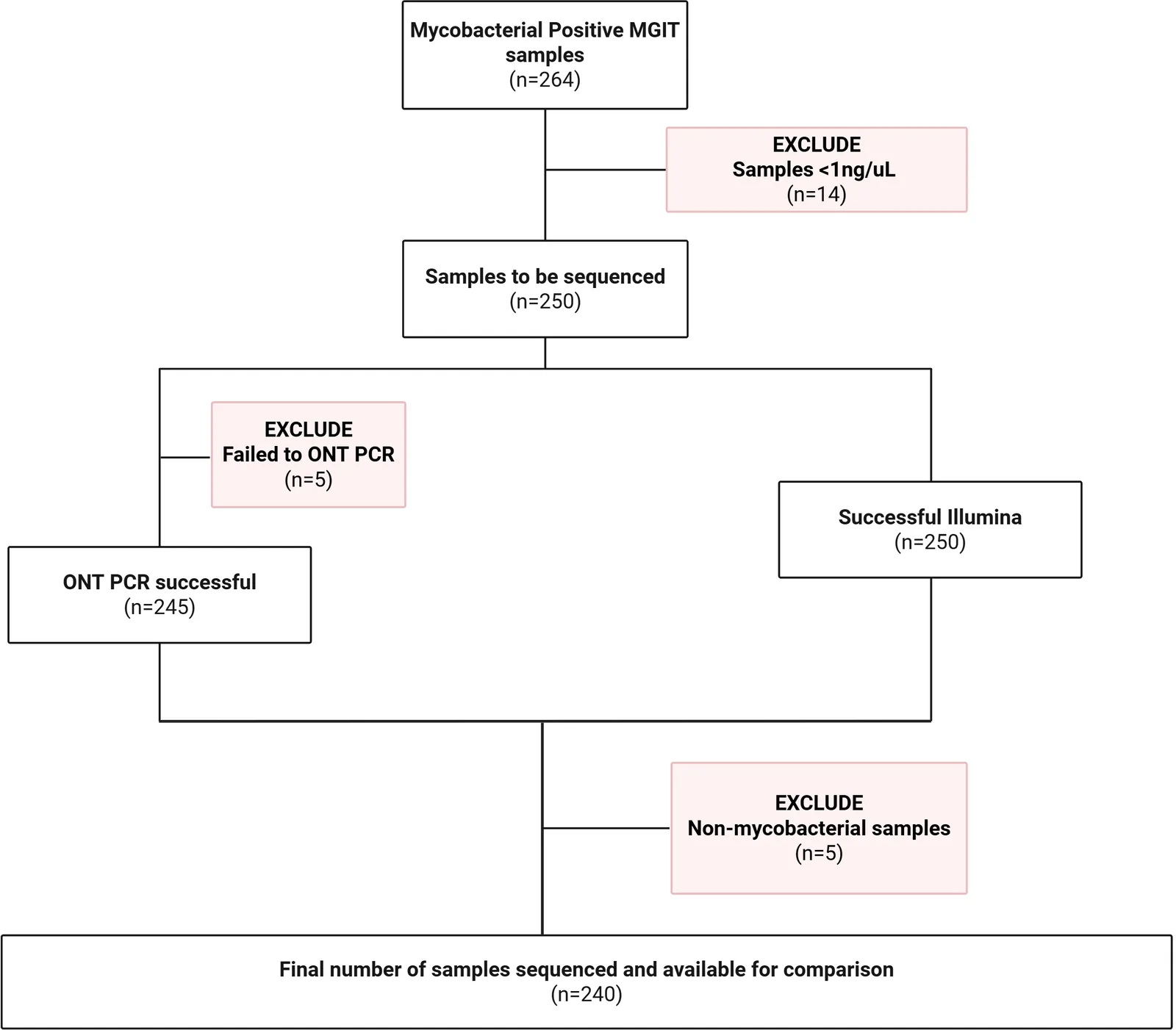
Flow diagram of sample inclusion. Of 264 positive MGIT cultures, 240 were successfully sequenced on both Illumina and ONT platforms and classified as *Mycobacterium* spp. Fourteen samples were excluded because of insufficient DNA, five failed PCR amplification before ONT sequencing, and five were excluded because sequencing identified predominantly non-mycobacterial DNA.

Five additional samples were excluded because both sequencing platforms identified predominantly non-mycobacterial DNA (see Fig. 1). The predominant taxonomic assignments were *Homo sapiens*, *Rhodococcus*, *Pseudomonas aeruginosa*, *Streptococcus*, and *Gordonia polyisoprenivorans*. Although these MGIT cultures had been reported as AFB positive, mycobacterial DNA was detected only at very low abundance (all <0.5 × mean depth according to Sylph), precluding reliable genomic characterization.

The median sequence yield per sample was 1,342 Mbp for Illumina and 637 Mbp for ONT. For ONT, the median N50 was 3,865 bp, and the median mean read quality score was 16.6 (Fig. S1 to S4).

### Comparison of species between ONT and Illumina

Of the 240 mycobacterial samples sequenced by both Illumina and ONT, species assignment was concordant for 236/240 (98.3%, 95% CI: 95.8%–99.5%) samples. All samples had at least one *Mycobacterium* species identified by both platforms, and a total of 21 distinct species were detected.

In three samples, Sylph was unable to assign a species. However, competitive mapping against mycobacterial reference genomes (see Materials and Methods) demonstrated 50%–70% genome coverage of *M. gordonae*/*M. paragordonae* in both platforms.

Mixed-species infections were identified in 30 samples by at least one platform. In 26 of these, the same combination of species was consistently detected by both platforms. For these concordant mixed samples, the ratio of Illumina to ONT relative abundance had a median of 0.99 (IQR: 0.93–1.36, Fig. S7).

In the remaining four samples, an additional mycobacterial species was detected by only one platform (Illumina, *n* = 3; ONT, *n* = 1). In two cases, the discordant species was present at low abundance (<2%), whereas in the other two, it exceeded 30% abundance: *M. abscessus* was detected only by Illumina in one sample, and *M. tuberculosis* only by ONT in another.

These results demonstrate a high level of concordance between ONT and Illumina for species identification, including in mixed-species infections.

### Platform comparison of *M. tuberculosis* samples

A total of 46/240 (19.2%) samples were identified as *M. tuberculosis* by both ONT and Illumina. Four samples were excluded due to low read depth 3.2 (<10×) when sequenced by either Illumina (*n* = 3) or both platforms (*n* = 1), leaving 42 paired high-quality *M. tuberculosis* genomes for downstream analyses.

Identical sub-lineage assignments were obtained across platforms for all 42 samples. The isolates were phylogenetically diverse, with all four major lineages represented and a total of 20 distinct sub-lineages (Fig. S8) identified.

**Fig 2:**
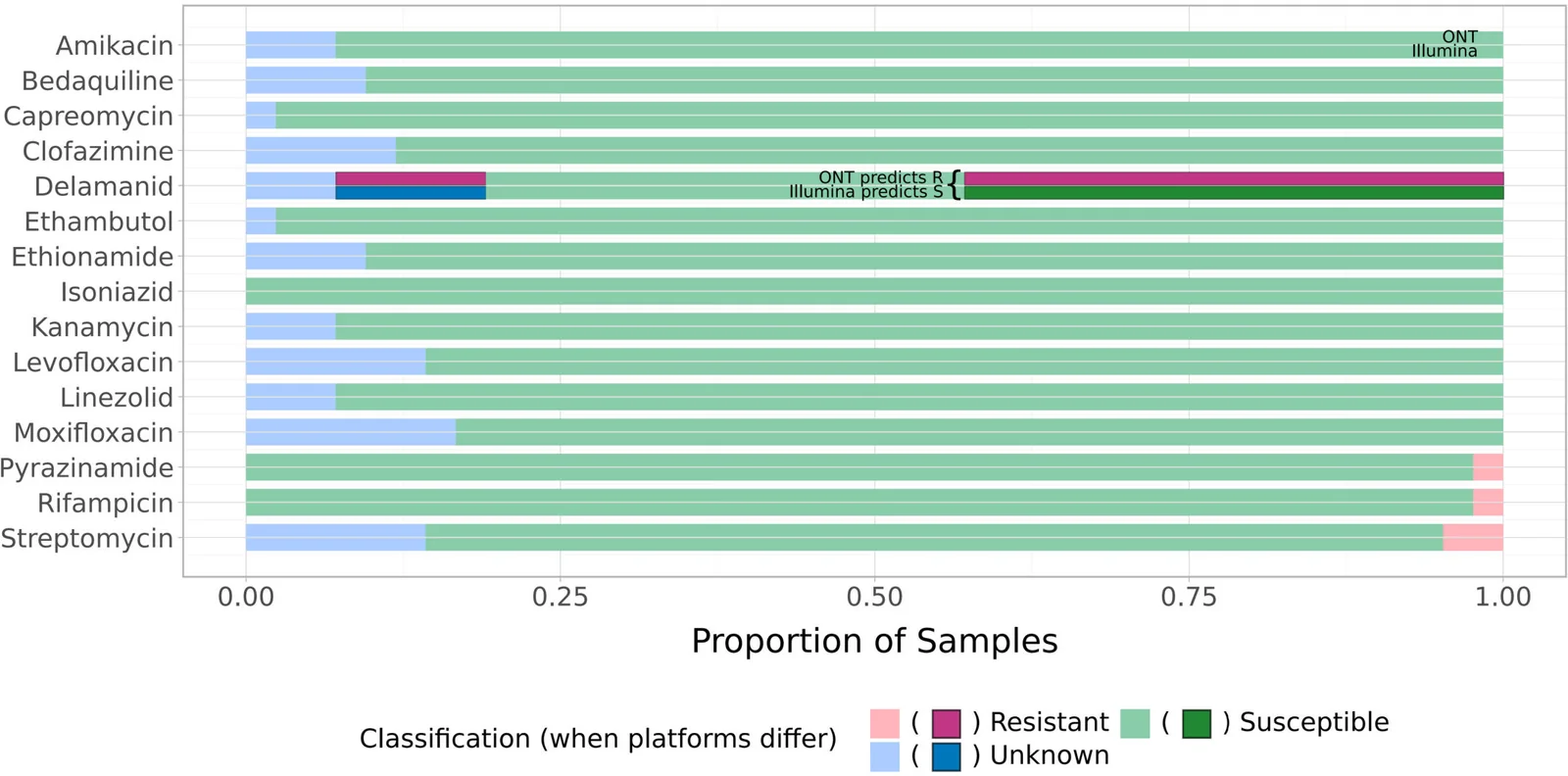
Comparison of genotypic resistance classification between ONT and Illumina. For each drug, the lower bar shows the proportions of samples classified as Unknown, Susceptible, or Resistant by Illumina, and the upper shows the corresponding classifications by ONT. Discordant classifications are highlighted by darker colors and bold outlines. The annotated delamanid segment indicates that 43% of samples classified as susceptible by Illumina were classified as resistant by ONT. Complete agreement was observed for all other drugs.

Very few mutations associated with antimicrobial resistance were detected in this data set. With the exception of delamanid, only four samples showed resistance to the 15 drugs covered by WHOv2 (Fig. 2). In three samples, the detected mutation was identical in both platforms, but one sample had two mutations conferring resistance to the same drug: one identical across platforms and one unique to Illumina (a minor SNP with 15% read support). A total of 23/42 (54.8%) of the ONT genomes were classified as resistant to delamanid due to a 62 bp deletion that starts seven bases before the end of *fbiC*. According to WHOv2, this deletion triggers a loss-of-function rule resulting in a “Resistant” call. However, this region corresponds to a tandem repeat downstream of *fbiC*, and the deletion is not expected to affect the primary coding sequence (Supplemental section 7.2).

All samples had sufficient depth at all genetic loci associated with resistance (group 1) according to WHOv2, and no samples returned a “Fail” result for any drugs. The pipeline returned an “Unknown” classification for uncatalogued mutations in resistance genes. These calls were entirely concordant between ONT and Illumina (38 mutations across 23 samples). A total of 417 mutations not associated with antimicrobial resistance were identified. Of these, 409/417 (98.1%) were detected by both platforms, while the remaining variants represented minority populations: five SNPs unique to Illumina, two SNPs unique to ONT, and one indel unique to ONT.

**Fig 3:**
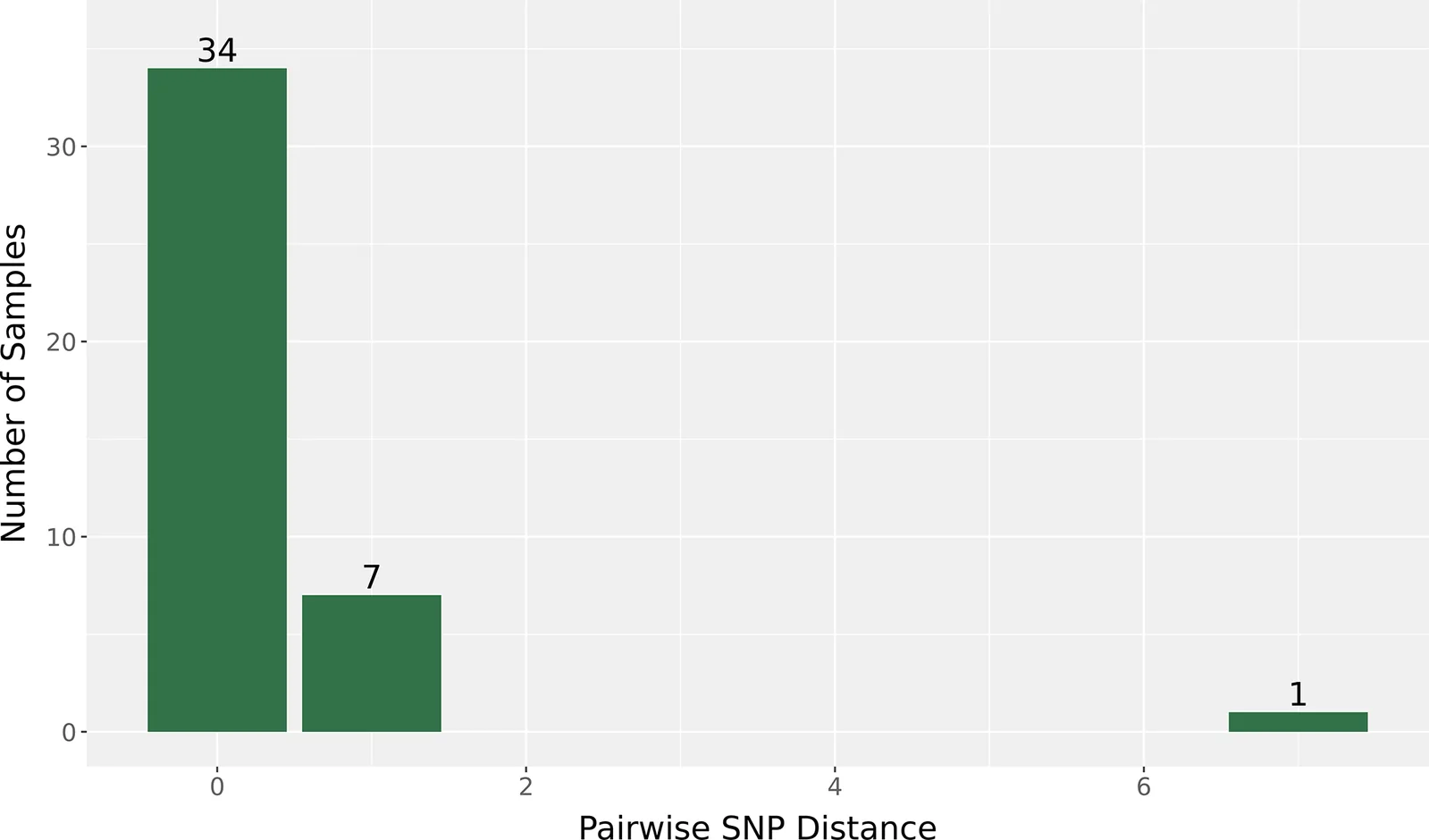
Pairwise SNP distances between Illumina and ONT sequencing of the same *Mycobacterium tuberculosis* samples. The bar chart shows the SNP distance for each of the 42 MTBC isolates sequenced on both platforms. A total of 34/42 (81.0%) of samples had identical SNP profiles (0 SNP differences).

Genomic similarity was assessed using pairwise SNP distances (see Supplemental section 7.3 for details of calculation and masking). Pairwise SNP distances between ONT and Illumina assemblies were calculated for each of the 42 *M. tuberculosis* samples (Fig. 3). The mean cross-platform SNP distance was 0.3, with 34/42 (81.0%) sample pairs showing no SNP differences and therefore being identical. The largest cross-platform SNP distance observed for a single isolate was seven SNPs.

Three pairs of samples were closely related (≤20 SNPs apart). Hospital records confirmed that, in each case, the paired samples were obtained from the same patient at different time points. The SNP distance between samples within each pair was zero, irrespective of the sequencing platform used.

For all pairs of samples, the absolute difference in SNP distance between platforms was calculated as a fraction of the Illumina distance (excluding the three zero-distance pairs described above). The median of this metric across all pairs was 0.7% or 0.8% if considering cross-platform comparisons (Supplemental section 7.3).

Overall, ONT and Illumina demonstrated highly concordant performance for lineage assignment, SNP-based relatedness, and resistance detection.

### Time to reliable species identification with ONT

**Fig 4:**
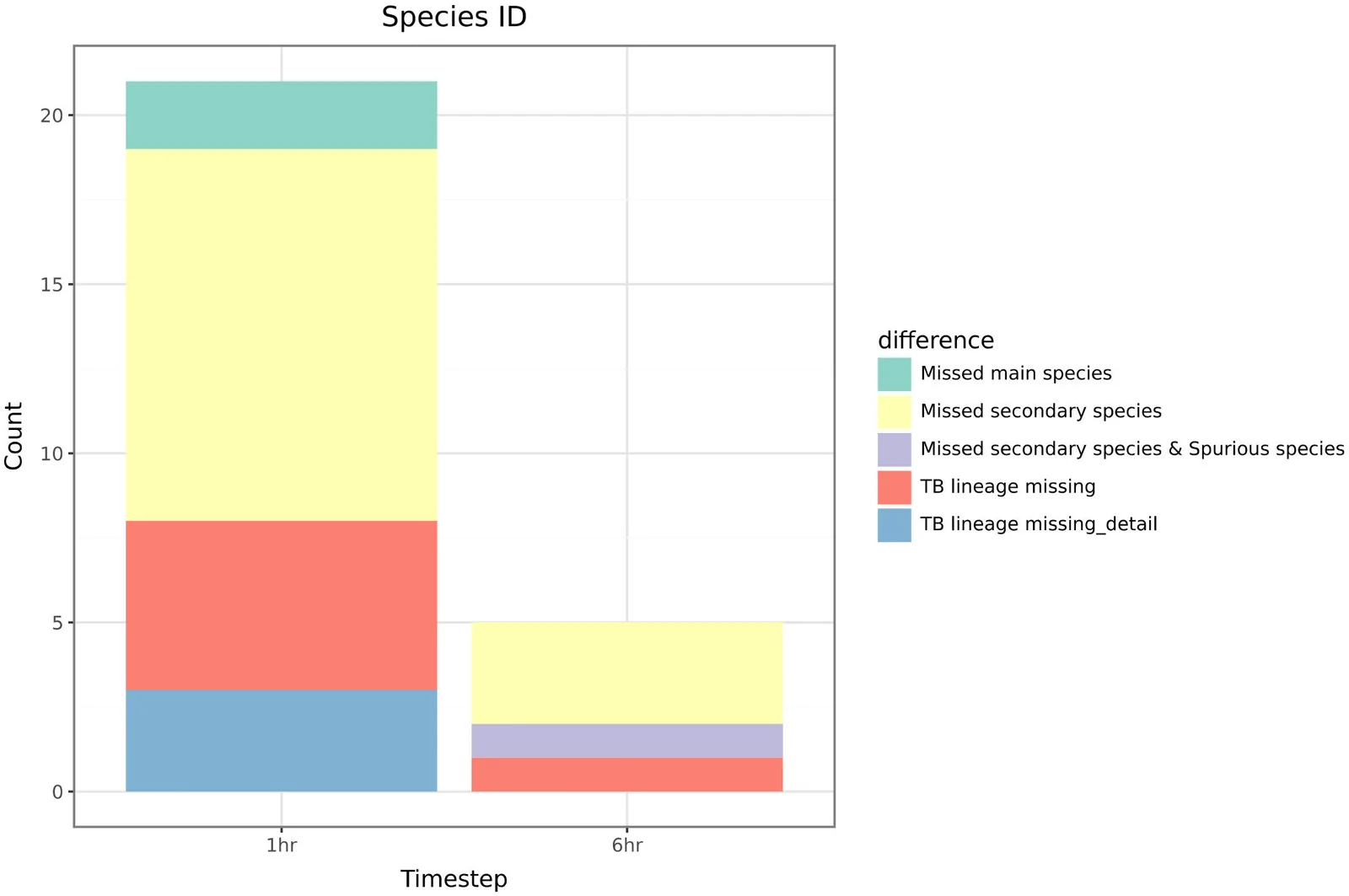
Discordance in species identification and lineage assignment at 1 and 6 h of ONT sequencing relative to the final 72-h result. At 1 h, 21/237 (8.9%) samples were discordant with the 72-h result, including two samples in which the predominant species was not identified. By 6 h, discordance had decreased to 5/237 (2.1%), with all remaining discordances representing minor differences.

To determine the earliest reliable time point for species identification, ONT sequencing data were analyzed after 1, 6, and 72 h. Subsampling of reads at 1 h and 6 h demonstrated high concordance with species assignments obtained from the final 72 h data set. Of the 240 samples, 237 had a species identified after 72 h. Of these, 224/237 (94.5%) were correctly identified after 1 h, increasing to 233/237 (98.3%) after 6 h (Fig. 4 and Fig. S10). The remaining four samples at 6 h differed only by the absence of low-abundance secondary species (<1%) that were detected after 72 h.

In two samples, no species was identified after 1 h due to insufficient reads, causing early pipeline termination. There were no instances of incorrect primary species assignments at 6 h, and only one secondary species was detected that was not present in the 72-h data set, which corresponded to a non-mycobacterial organism.

Lineage assignment for *M. tuberculosis* was performed using Mykrobe. After 1 h, eight samples showed discordance compared to the 72-h result, including missing lineage calls or reduced sub-lineage resolution. By 6 h, this was reduced to a single discordant sample (Fig. 4).

**Fig 5:**
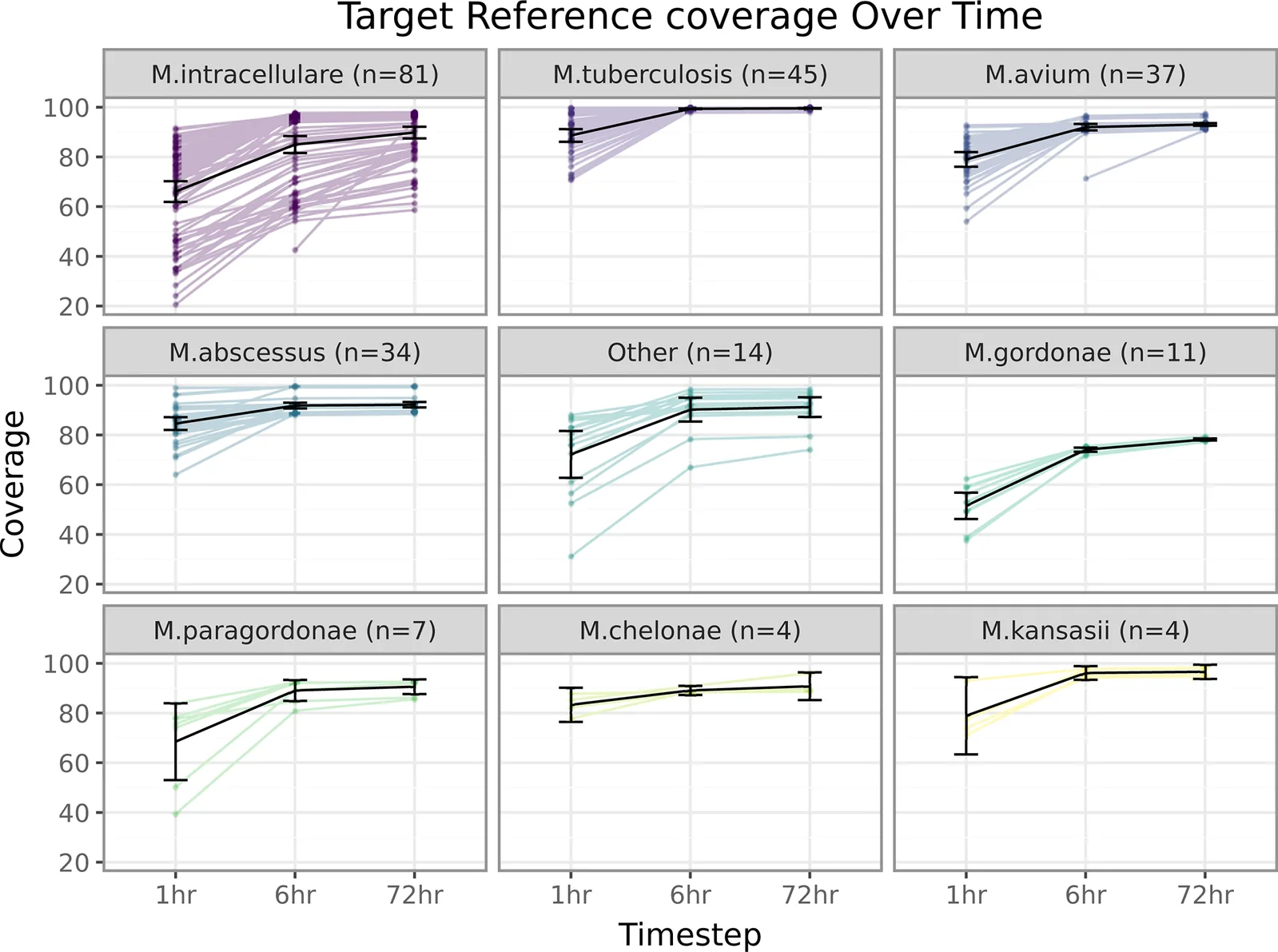
Genome coverage of the predominant *Mycobacterium* species after 1, 6, and 72 h of ONT sequencing. Genome coverage increased with sequencing time for most species. *M. gordonae* consistently showed lower genome coverage than other species, including after 72 h.

After 1 h, the mean number of reads was 5,610 (95% interval: 1,730–14,300) and mean read depth for the dominant species ranged from 0.1 to 10× (Fig. S11), with genome coverage varying between 20% and 100% (Fig. 5). After 6 h, total reads and mean read depth increased by approximately an order of magnitude, resulting in improved genome coverage. Further sequencing to 72 h increased read depth but provided minimal additional gains in genome coverage for the dominant species.

For *M. tuberculosis*, mean read depth increased from 21.1 ± 12.9 at 6 h to 142.9 ± 86.1 at 72 h. However, variability between samples indicates that while some can be fully analyzed after 6 h, others require longer sequencing to achieve sufficient depth for downstream analyses.

## DISCUSSION

This study demonstrates that a semi-automated DNA extraction and PCR-based ONT workflow provides a practical approach for routine genomic characterization of primary MGIT cultures. Unlike previous studies that have primarily evaluated ONT sequencing performance, our objective was to develop a workflow that could be integrated into routine clinical laboratory practice using readily available instrumentation and minimal additional hands-on time. The optimized workflow generated sufficient DNA for reliable ONT sequencing from primary MGIT cultures and, when compared with Illumina sequencing, achieved highly concordant *Mycobacterium* species identification, MTBC lineage assignment, antimicrobial resistance prediction, and SNP-based relatedness.

Compared with our previous extraction workflow, the modifications introduced to sample processing and heat inactivation were associated with an approximately 25-fold increase in median DNA concentration. Although this comparison was not performed head-to-head, the magnitude of improvement suggests that the optimized workflow substantially improved DNA recovery from primary MGIT cultures, increasing the proportion of samples approaching the DNA input recommended for PCR-free ONT library preparation while providing a more robust input for the PCR-based workflow used in this study. During workflow optimization, replacing LongAmp Hot Start Taq with Ex Premier DNA Polymerase produced more consistent PCR amplification and higher DNA yields. Although this modification was not formally evaluated within the present study, it contributed to the robustness of the workflow and may be beneficial for laboratories sequencing low-input clinical specimens.

The high proportion of samples falling below the recommended DNA input for the Rapid Barcoding Kit further supports the use of PCR-based amplification for MGIT-derived DNA, where yield and purity are frequently limiting. In this study, a pragmatic internal threshold of 1 ng/μL was used. However, the Rapid PCR Barcoding workflow supports substantially lower DNA inputs (1 ng in 3 μL, approximately 0.33 ng/μL), and six samples with DNA concentrations below the study threshold were successfully sequenced using this approach, demonstrating that reliable ONT sequencing is feasible at lower DNA concentrations (Table S2). Together, these optimizations overcome a major practical barrier to implementing ONT sequencing from primary MGIT cultures.

A key advantage of this workflow is that it was specifically designed to support implementation within routine diagnostic laboratories rather than specialist sequencing centers. By combining semi-automated DNA extraction, PCR-based library preparation, and ONT sequencing, genomic characterization can be performed locally from positive MGIT cultures, reducing reliance on referral to external sequencing services and providing the potential for shorter turnaround times.

An additional advantage of ONT sequencing is continuous data generation, allowing run metrics to be monitored in real time. Subsampling analyses showed that reliable species identification was achievable after 6 h for 98.3% of samples, although longer sequencing was required to achieve sufficient genome coverage for antimicrobial resistance prediction in some isolates. These findings indicate that the sequencing duration required depends on the intended clinical application, with shorter runs sufficient for species identification and longer runs required for comprehensive genomic characterization.

Despite recent progress, ONT reads still have higher error rates than Illumina reads. In our data set, the discrepant SNPs in ONT data clustered within discrete genomic regions rather than being randomly distributed. This pattern suggests that selective masking or region-aware filtering could improve cross-platform concordance, particularly for phylogenetic applications. Importantly, the magnitude of disagreement outside these regions was small, indicating that ONT-derived genomes are sufficiently accurate for species identification, lineage assignment, antimicrobial resistance prediction, and transmission analysis. Continued bioinformatic refinements, particularly to variant calling, are likely to further enhance cross-platform reproducibility, enabling reliable comparison of results between laboratories using different sequencing technologies, consistent with findings from recent comparative studies (12).

Two samples showed evidence of mixed mycobacterial species. In one case, *M. tuberculosis* was detected by ONT but not by Illumina, whereas in the other, *M. abscessus* was detected by Illumina but not by ONT. These discrepancies are more likely to reflect biological heterogeneity or sample-level contamination than systematic differences between sequencing platforms. Importantly, these events were rare and had minimal impact on the overall concordance between platforms.

A subset of Illumina data sets contained >5% non-mycobacterial reads by Sylph taxonomic profiling after Kraken2 filtering. This likely reflects differences in how short and long reads interact with taxonomic classifiers. Longer ONT reads provide greater genomic context and therefore are more confidently assigned by tools such as Kraken2, whereas shorter Illumina reads are more frequently left unclassified rather than misclassified. These unclassified reads are subsequently assigned low-confidence labels by downstream tools such as Sylph in our pipeline, inflating the apparent proportion of non-mycobacterial sequences. This highlights the need for harmonized, platform-aware taxonomic approaches when comparing sequencing modalities.

This study has several limitations. It was conducted at a single center using MGIT-positive cultures rather than primary clinical specimens and therefore evaluates downstream genomic characterization rather than primary diagnosis. Although ONT sequencing was performed in-house, Illumina sequencing was undertaken by a commercial provider, introducing methodological differences that may have influenced the comparison. Resistance prediction for non-tuberculous mycobacteria was not evaluated, and the *M. tuberculosis* data set contained limited antimicrobial resistance diversity, although all WHO group 1 resistance-associated loci were adequately covered. A small proportion of samples (5/250; 2%) failed PCR amplification, highlighting the potential need for repeat extraction or alternative amplification strategies for challenging specimens. We compared ONT sequencing results with those generated by Illumina rather than independent laboratory reference methods, limiting our ability to resolve discordant mixed-species identifications. Finally, variability in ONT sequencing performance between flow cells, including one run with reduced throughput and read quality, emphasizes the importance of appropriate run-level quality control. Further evaluation in larger, multicenter cohorts will be required before routine diagnostic implementation.

In conclusion, by overcoming the challenges associated with low-yield MGIT-derived DNA, the workflow described here provides a practical route toward routine in-house genomic characterization of mycobacterial cultures within hospital diagnostic laboratories. Its high concordance with Illumina sequencing for species identification, MTBC lineage assignment, antimicrobial resistance prediction, and SNP-based relatedness supports wider implementation of ONT sequencing in clinical mycobacteriology.

## Supplementary Material

Reviewer comments

## Data Availability

Reads have been deposited in the ENA under project code PRJEB106303. The GitHub repository containing the code and supporting materials used for the study is publicly available at: https://github.com/oxfordmmm/ouh_mgit_study.

## SUPPLEMENTAL MATERIAL

Supplemental text spectrum.01402-26-s0001.pdf Supplemental text.

Table S1 spectrum.01402-26-s0002.csv Mapping references.

Table S2 spectrum.01402-26-s0003.xlsx combined summary species ID.

Table S3 spectrum.01402-26-s0004.xlsx Resistance gDST.

Table S4 spectrum.01402-26-s0005.xlsx All detected mutations in resistance genes.

Table S5 spectrum.01402-26-s0006.xlsx SNP distances between platforms on the same sample.

Table S6 spectrum.01402-26-s0007.xlsx SNP distance between all pairs of samples.

Table S7 spectrum.01402-26-s0008.xlsx Sylph species counts.

Table S8 spectrum.01402-26-s0009.xlsx Competitive mapping.

OPEN PEER REVIEW reviewer-comments.pdf An accounting of the reviewer comments and feedback.

ASM does not own the copyrights to Supplemental Material that may be linked to, or accessed through, an article. The authors have granted ASM a non-exclusive, world-wide license to publish the Supplemental Material files. Please contact the corresponding author directly for reuse.
