# Supplementary material for: Validation of an optimized Oxford Nanopore sequencing workflow versus Illumina for mycobacteria from primary MGIT culture
Source: Microbiol Spectr. 2026 Aug 20;14(10):e01402-26. doi: 10.1128/spectrum.01402-26 (PMC13689354; doi:10.1128/spectrum.01402-26)
Supplement: Supplementary file 1 [file reviewer-comments.pdf]

ASM | Microbiology Spectrum

Validation of an optimised Oxford Nanopore sequencing workflow versus Illumina for mycobacteria from primary MGIT culture

Catriona Baker, Matthew Colpus, Jessica Gentry, Alexandra Hall, Eloïse Roghi, Hermione Webster, Bronte Drummond, Ruth Cooper, Hieu Thai, Jeremy Westhead, Robert Turner, Tim Peto, Philip Fowler, Marcus Morgan, and Derrick Crook

Corresponding Author(s): Catriona Baker, University of Oxford Nuffield Department of Medicine

**Review Timeline:**		
	Submission Date:	May 6, 2026
	Editorial Decision:	June 1, 2026
	Revision Received:	July 28, 2026
	Accepted:	July 30, 2026

Editor: Paul Luethy

Reviewer(s): The reviewers have opted to remain anonymous.

Transaction Report:

(Note: With the exception of the correction of typographical or spelling errors that could be a source of ambiguity, letters and reports are not edited. The original formatting of letters and referee reports may not be reflected in this compilation.)

DOI: https://doi.org/10.1128/spectrum.01402-26

Re: Spectrum01402-26 (Validation of an optimised Oxford Nanopore sequencing workflow versus Illumina for mycobacteria from primary MGIT culture)

Dear Dr. Derrick W Crook:

Thank you for the privilege of reviewing your work. Below you will find my comments, instructions from the Spectrum editorial office, and the reviewer comments.

Please return the manuscript within 60 days; if you cannot complete the modification within this time period, please contact me. If you do not wish to modify the manuscript and prefer to submit it to another journal, notify me immediately so that the manuscript may be formally withdrawn from consideration by Spectrum.

Revision Guidelines
To submit your modified manuscript, log into the submission site at https://spectrum.msubmit.net/cgi-bin/main.plex. Go to Author Tasks and click the appropriate manuscript title to begin. The information you entered when you first submitted the paper will be displayed; update this as necessary. Note the following requirements:

• Upload point-by-point responses to the issues raised by the reviewers in a file named "Response to Reviewers," NOT in your cover letter.
• Upload a compare copy of the manuscript (without figures) as a "Marked-Up Manuscript" file.
• Upload a clean .DOC/.DOCX version of the revised manuscript and remove the previous version.
• Each figure must be uploaded as a separate, editable, high-resolution file (TIFF or EPS preferred), and any multipanel figures must be assembled into one file.
• Any supplemental material intended for posting by ASM should be uploaded with their legends separate from the main manuscript. You can combine all supplemental material into one file (preferred) or split it into a maximum of 10 files with all associated legends included.

For complete guidelines on revision requirements, see our Submission and Review Process webpage. Submission of a paper that does not conform to guidelines may delay acceptance of your manuscript.

Data availability: ASM policy requires that data be available to the public upon online posting of the article, so please verify all links to sequence records, if present, and make sure that each number retrieves the full record of the data. If a new accession number is not linked or a link is broken, provide Spectrum production staff with the correct URL for the record. If the accession numbers for new data are not publicly accessible before the expected online posting of the article, publication may be delayed; please contact production staff (Spectrum@asmusa.org) immediately with the expected release date.

Publication Fees: For information on publication fees and which article types are subject to charges, visit our website. If your manuscript is accepted for publication and any fees apply, you will be contacted separately about payment during the production process; please follow the instructions in that e-mail. Arrangements for payment must be made before your article is published.

ASM Membership: Corresponding authors may join or renew ASM membership to obtain discounts on publication fees. Need to upgrade your membership level? Please contact Customer Service at Service@asmusa.org.

The ASM Journals program strives for constant improvement in our submission and publication process. Please tell us how we can improve your experience by taking this quick Author Survey.

Thank you for submitting your paper to Spectrum.

Sincerely,
Paul Luethy
Editor
Microbiology Spectrum

Reviewer #1 (Comments for the Author):

Major Comments

Results Section "ONT Sequencing After 1, 6, and 72 hours" - The authors provide an analysis of the minimal time needed for ONT sequencing to generate reliable species identification. While this is an important factor in turnaround time, in practice this

will be influenced by the number of available/functional pores in the flow cell. The authors should also provide information on the number of reads (in a range) that were generated at these time points, in addition to the mean depth information that is already provided.

Figure 4 - The data in this figure and the figure legend do not appear to be the same. The figure summarizes a count of samples with missing identifications/lineage assignments, while the figure legend describes the concordant samples. The authors should provide either an appropriate figure legend for the data presented, or revise the data in the figure.

Discussion/Limitations - The authors conclude that their workflow resulted in "accurate species identification" (lines 364-365), however, their methods compared one sequencing method to another, and not to routinely used and accepted methods for mycobacterial identification. Since the authors did not compare to the standard of care culture results from these positive MGIT broths, this should be identified as a limitation of their study.

Minor Comments

Line 73 - The acronym "MALDI-TOF" should be defined when first used.

Line 75 (and throughout manuscript) - "spp." should not be italicized.

Line 207 - "MMM" should be defined.

Lines 210-211 - The authors state that either Hostile or Deacon were used to remove human reads from samples. They should specify how they determined which one to use for a given specimen.

Table 2 - This table would benefit from the addition of the actual numbers of specimen types, rather than just the proportion.

Lines 257-260 - The authors state that 5 samples were excluded from analysis for having non-mycobacterial sequencing results, however, the methods state that all samples were confirmed to be AFB positive by Ziehl-Neelsen staining. For some organisms (e.g. Gordonia) this may not be discrepant, but for others (e.g. Pseudomonas) this is discrepant. The authors should comment on these discrepancies and whether these samples were truly positive for mycobacterial species by culture.

Reviewer #3 (Comments for the Author):

Thank you for the invitation to review this manuscript entitled "Validation of an optimised Oxford Nanopore 2 sequencing workflow versus Illumina for mycobacteria from primary MGIT culture". The data presented by Baker et al. outlines an example of a sequencing workflow that addresses some key challenges (e.g., low DNA yields, hands-on time) in mycobacterial sequencing.

Major Comments
1. The authors frequently mention that a key outcome of this work was an "optimized" DNA extraction method. Do you the authors have any data on how they arrived at this optimized extraction method (i.e., comparison of DNA yield to other extraction methods or protocol versions)? If not, I suggest softening this conclusion particularly given that the extraction yields were still low and required use of a PCR-based barcoding kit.
2. Line 286: The authors stated that four samples were excluded due to low-reads / depth. What was the cut-off for samples considered low-read depth and was this applied consistently?
3. The results section focuses on sequencing data obtained over time, but the per sample data output per hour will also vary based on the number of samples batched onto each flow cell. Could the authors please state how many samples were batched onto each R10 flow cell run?
4. Line 323: The authors state that there were some samples that showed close SNP distances. Do the authors have access to any epidemiological metadata to reinforce these findings?
5. Line 382: The authors did not test samples below 1 ng/uL, so outside of the manufacturer's recommendations cited there is not data presented supporting that lower input concentrations of MTBC DNA would be successful. I recommend changing "indicating" to "suggesting" to soften this conclusion.
6. Line 396: As an alternative explanation, I suggest highlighting the limitations (sensitivity) in the setting of MGIT cultures that are positive for multiple organisms. Do the authors have other ways to investigate whether both organisms were truly present in these samples (i.e., plating to solid media, PCR testing, etc.)

Minor Comments
1. Figure 2: The "upper" and "lower" bards are difficult to appreciate. Could there be a more pronounced line separating the two? This figure could also benefit from a scale on the X-axis that shows %.
2. There is a very large number of supplemental figures (11) with large blocks of text. I recommend combining figures into panels, removing if not necessary to support conclusions, and/or relocating them to be panels in the main text.

Major Comments

Results Section “ONT Sequencing After 1, 6, and 72 hours” – The authors provide an analysis of the minimal time needed for ONT sequencing to generate reliable species identification. While this is an important factor in turnaround time, in practice this will be influenced by the number of available/functional pores in the flow cell. The authors should also provide information on the number of reads (in a range) that were generated at these time points, in addition to the mean depth information that is already provided.

Figure 4 – The data in this figure and the figure legend do not appear to be the same. The figure summarizes a count of samples with missing identifications/lineage assignments, while the figure legend describes the concordant samples. The authors should provide either an appropriate figure legend for the data presented, or revise the data in the figure.

Discussion/Limitations – The authors conclude that their workflow resulted in “accurate species identification” (lines 364-365), however, their methods compared one sequencing method to another, and not to routinely used and accepted methods for mycobacterial identification. Since the authors did not compare to the standard of care culture results from these positive MGIT broths, this should be identified as a limitation of their study.

Minor Comments

Line 73 – The acronym “MALDI-TOF” should be defined when first used.

Line 75 (and throughout manuscript) – “spp.” should not be italicized.

Line 207 - “MMM” should be defined.

Lines 210-211 – The authors state that either Hostile or Deacon were used to remove human reads from samples. They should specify how they determined which one to use for a given specimen.

Table 2 – This table would benefit from the addition of the actual numbers of specimen types, rather than just the proportion.

Lines 257-260 – The authors state that 5 samples were excluded from analysis for having non-mycobacterial sequencing results, however, the methods state that all samples were confirmed to be AFB positive by Ziehl-Neelsen staining. For some organisms (e.g. Gordonia) this may not be discrepant, but for others (e.g. Pseudomonas) this is discrepant. The authors should comment on these discrepancies and whether these samples were truly positive for mycobacterial species by culture.

Thank you for the invitation to review this manuscript entitled “Validation of an optimised Oxford Nanopore 2 sequencing workflow versus Illumina for mycobacteria from primary MGIT culture”. The data presented by Baker et al. outlines an example of a sequencing workflow that addresses some key challenges (e.g., low DNA yields, hands-on time) in mycobacterial sequencing.

Major Comments

1. The authors frequently mention that a key outcome of this work was an “optimized” DNA extraction method. Do you the authors have any data on how they arrived at this optimized extraction method (i.e., comparison of DNA yield to other extraction methods or protocol versions)? If not, I suggest softening this conclusion particularly given that the extraction yields were still low and required use of a PCR-based barcoding kit.
2. Line 286: The authors stated that four samples were excluded due to low-reads / depth. What was the cut-off for samples considered low-read depth and was this applied consistently?
3. The results section focuses on sequencing data obtained over time, but the per sample data output per hour will also vary based on the number of samples batched onto each flow cell. Could the authors please state how many samples were batched onto each R10 flow cell run?
4. Line 323: The authors state that there were some samples that showed close SNP distances. Do the authors have access to any epidemiological metadata to reinforce these findings?
5. Line 382: The authors did not test samples below 1 ng/uL, so outside of the manufacturer’s recommendations cited there is not data presented supporting that lower input concentrations of MTBC DNA would be successful. I recommend changing “indicating” to “suggesting” to soften this conclusion.
6. Line 396: As an alternative explanation, I suggest highlighting the limitations (sensitivity) in the setting of MGIT cultures that are positive for multiple organisms. Do the authors have other ways to investigate whether both organisms were truly present in these samples (i.e., plating to solid media, PCR testing, etc.)

Minor Comments

1. Figure 2: The “upper” and “lower” bards are difficult to appreciate. Could there be a more pronounced line separating the two? This figure could also benefit from a scale on the X-axis that shows %.
2. There is a very large number of supplemental figures (11) with large blocks of text. I recommend combining figures into panels, removing if not necessary to support conclusions, and/or relocating them to be panels in the main text.

Title: Validation of an optimised Oxford Nanopore sequencing workflow versus Illumina for mycobacteria from primary MGIT culture
Authors: Baker CS, Colpus M, Gentry J, Hall A, Roghi E, Webster H, Drummond B, Cooper R, Thai H, Westhead J, Turner R, Peto TEA, Fowler PW, Morgan M, Crook DW
Your reference: Spectrum01402-26

We thank the reviewers for their constructive feedback which has, we hope, improved the clarity and scope of this manuscript. To aid with cross-referencing we have numbered all the points made by each reviewer (e.g. §2.3) and then where changes have been made to the manuscript the number is annotated in the margin in red. Our responses are in *italics*.

Reviewer #1

Major Comments

1.1 Results Section "ONT Sequencing After 1, 6, and 72 hours" - The authors provide an analysis of the minimal time needed for ONT sequencing to generate reliable species identification. While this is an important factor in turnaround time, in practice this will be influenced by the number of available/functional pores in the flow cell. The authors should also provide information on the number of reads (in a range) that were generated at these time points, in addition to the mean depth information that is already provided.

We agree: the variability in ONT output is due in part to the number of active sequencing pores. We only used flow cells with a pore number of over 800 (i.e. within ONT warranty) and the average across the 12 runs was 1,356 pores before loading.

We have added to the manuscript text the mean number of reads and also an additional sub-figure to the Supplement. Given the variable length of ONT reads we felt mean depth was more informative to most readers, hence why we have only

included the new graph in the supplement.

1.2 Figure 4 - The data in this figure and the figure legend do not appear to be the same. The figure summarizes a count of samples with missing identifications/lineage assignments, while the figure legend describes the concordant samples. The authors should provide either an appropriate figure legend for the data presented, or revise the data in the figure.

Thank you for pointing this out. We have changed the figure legend to more appropriately represent what can be seen in the figure.

1.3 Discussion/Limitations - The authors conclude that their workflow resulted in "accurate species identification" (lines 364-365), however, their methods compared one sequencing method to another, and not to routinely used and accepted methods for mycobacterial identification. Since the authors did not compare to the standard of care culture results from these positive MGIT broths, this should be identified as a limitation of their study.

The reviewer is correct – we have not compared against culture-based laboratory methods and we have therefore added this as a limitation in the discussion as suggested. However, within the UK at least the reference standard method for NTM species identification by UKHSA is currently genetic (via Illumina sequencing and the COMPASS pipeline). Although we are not using the COMPASS pipeline directly, Illumina sequencing in the UK would hence be considered the standard method of species identification.

Minor Comments

1.4 Line 73 - The acronym "MALDI-TOF" should be defined when first used.

Thank you, this correction has now been made.

1.5 Line 75 (and throughout manuscript) - "spp." should not be italicized.

Thank you, this correction has now been made.

1.6 Line 207 - “MMM” should be defined.

Thank you, this correction has now been made.

1.7 Lines 210-211 - The authors state that either Hostile or Deacon were used to remove human reads from samples. They should specify how they determined which one to use for a given specimen.

We used Hostile for the earlier runs to remove human reads. Both Hostile and Deacon are written by the same bioinformatician with Deacon superceding Hostile. He decided to give the new version a new name because it was re-written in a different programming language (Rust) to speed up processing. Hence in our hands they perform equivalently, the main difference is speed. Slight differences in human read removal are extremely unlikely to impact our results but are an important privacy consideration so we do not consider this an important factor.

1.8 Table 2 - This table would benefit from the addition of the actual numbers of specimen types, rather than just the proportion.

Thank you for pointing this out, we have added to Table 2 the number alongside the proportion for clarification.

1.9 Lines 257-260 - The authors state that 5 samples were excluded from analysis for having non-mycobacterial sequencing results, however, the methods state that all samples were confirmed to be AFB positive by Ziehl-Neelsen staining. For some organisms (e.g. Gordonia) this may not be discrepant, but for others (e.g. Pseudomonas) this is discrepant. The authors should comment on these discrepancies and whether these samples were truly positive for mycobacterial species by culture.

Thank you for highlighting this important point. All five samples were Ziehl–Neelsen positive and originated from MGIT cultures that had been reported as positive for acid-fast bacilli. However, sequencing identified only very low levels of mycobacterial DNA (all ¡0.5× mean depth according to Sylph), with the majority of reads assigned to non-mycobacterial organisms. These samples were therefore excluded from the comparative analyses because insufficient mycobacterial sequence data were available for reliable genomic characterisation. Unfortunately, the samples

had been heat-inactivated as part of the extraction workflow, precluding repeat culture or further microbiological investigation to determine whether these findings reflected contamination, mixed culture, or very low-abundance mycobacteria. We have clarified this point in the Results section.

Reviewer #2

No comments received.

Reviewer #3

Thank you for the invitation to review this manuscript entitled "Validation of an optimised Oxford Nanopore 2 sequencing workflow versus Illumina for mycobacteria from primary MGIT culture". The data presented by Baker et al. outlines an example of a sequencing workflow that addresses some key challenges (e.g., low DNA yields, hands-on time) in mycobacterial sequencing.

Major Comments

3.1. The authors frequently mention that a key outcome of this work was an "optimized" DNA extraction method. Do you the authors have any data on how they arrived at this optimized extraction method (i.e., comparison of DNA yield to other extraction methods or protocol versions)? If not, I suggest softening this conclusion particularly given that the extraction yields were still low and required use of a PCR-based barcoding kit.

We thank the reviewer for this suggestion. We agree that the original manuscript did not provide evidence supporting the use of the term "optimised". We have now added a comparison with our previous implementation of the George et al. 2020 MGIT extraction workflow, which yielded a median DNA concentration of 0.5 ng/μL across 154 MGIT cultures. In contrast, the modified heat inactivation and extraction protocol used in the current study yielded a median concentration of 12.4 ng/μL across 264 MGIT cultures. We have added these data to the Results

and Discussion and revised the text to clarify that this comparison is based on sequential evaluations rather than a contemporaneous head-to-head study. We have also added the comparison work that compared LongAMP Taq to Takara Ex Premiere that led to us incorporating Ex Premiere into our workflow

3.2. Line 286: The authors stated that four samples were excluded due to low-reads / depth. What was the cut-off for samples considered low-read depth and was this applied consistently?

We used a 10× read-depth cut off for both ONT and Illumina. This has now been added to the text.

3.3. The results section focuses on sequencing data obtained over time, but the per sample data output per hour will also vary based on the number of samples batched onto each flow cell. Could the authors please state how many samples were batched onto each R10 flow cell run?

That is a very good point, thank you. We have now added a line in the method section under ONT to show samples were multiplexed and the number of samples on each flow cell.

3.4. Line 323: The authors state that there were some samples that showed close SNP distances. Do the authors have access to any epidemiological metadata to reinforce these findings?

There were three pairs of samples which closely related as defined as being within 20 or fewer SNPs of each other. This is a conservative threshold and in each of the three cases OUH confirmed that both samples came from the same patient but were taken at different time points. We found no other cases of closely related samples. The next most related pair had a SNP distance over 60 which is well over standard thresholds. We've updated the text to clarify this.

3.5. Line 382: The authors did not test samples below 1 ng/uL, so outside of the manufacturer's recommendations cited there is not data presented supporting that lower input concentrations of MTBC DNA would be successful. I recommend changing "indicating" to "suggesting" to soften this conclusion.

We thank the reviewer for this comment. We have added data from six additional samples with DNA concentrations below 1 ng/µL that were successfully sequenced using the ONT workflow. The results are described in Supplementary Table S2 and are referenced in the Results and Discussion. We agree that the original statement was unsupported by data from the study cohort and have revised the text accordingly.

3.6. Line 396: As an alternative explanation, I suggest highlighting the limitations (sensitivity) in the setting of MGIT cultures that are positive for multiple organisms. Do the authors have other ways to investigate whether both organisms were truly present in these samples (i.e., plating to solid media, PCR testing, etc.)

We thank the reviewer for this suggestion. We agree that the interpretation of mixed-species detections in MGIT-positive cultures is limited by the absence of orthogonal confirmatory testing (and have added this to the discussion). At this point we sadly don't have the resources to investigate further. The discrepancy between ONT and Illumina on the two samples with high abundance secondary species we found very surprising and incongruent with the high concordance seen in the majority of mixed samples. Given ONT and Illumina sequence only divergence at the final stages (after growth and DNA extraction), it seems highly unlikely that both organisms were truly present. However, the sensitivity in MGIT cultures for multiple organisms is absolutely an issue, but it should apply to ONT and Illumina.

Minor Comments

3.7. Figure 2: The "upper" and "lower" bards are difficult to appreciate. Could there be a more pronounced line separating the two? This figure could also benefit from a scale on the X-axis that shows %.

We have added a percentage scale to the x-axis as suggested. Regarding the separation between the upper and lower bars, the absence of a dividing line where the classifications were identical was intentional, as it emphasises the few discordant results (limited to delamanid). We found that adding a separating line throughout reduced the visual impact of these differences. If the Editor or Reviewer feels that

additional separation would improve clarity, we would be happy to revise the figure accordingly.

3.8 There is a very large number of supplemental figures (11) with large blocks of text. I recommend combining figures into panels, removing if not necessary to support conclusions, and/or relocating them to be panels in the main text.

We appreciate the reviewer's comment and have reviewed the supplementary material. We believe that each figure provides information that supports the transparency and reproducibility of the study, while avoiding interruption of the flow of the main manuscript. As there are no space constraints in the supplementary material, we have retained the figures individually rather than combining them into panels, as this preserves their readability. We have, however, reviewed the supplementary figures to ensure that no unnecessary material has been included.

Re: Spectrum01402-26R1 (Validation of an optimised Oxford Nanopore sequencing workflow versus Illumina for mycobacteria from primary MGIT culture)

Dear Dr. Catriona S Baker:

Your manuscript has been accepted, and I am forwarding it to the ASM production staff for publication. Your paper will first be checked to make sure all elements meet the technical requirements. ASM staff will contact you if anything needs to be revised before copyediting and production can begin. Otherwise, you will be notified when your proofs are ready to be viewed.

Data Availability: ASM policy requires that data be available to the public upon online posting of the article, so please verify all links to sequence records, if present, and make sure that each number retrieves the full record of the data. If a new accession number is not linked or a link is broken, provide production staff with the correct URL for the record. If the accession numbers for new data are not publicly accessible before the expected online posting of the article, publication may be delayed; please contact ASM production staff immediately with the expected release date.

Publication Fees: For information on publication fees and which article types have charges, please visit our website. We have partnered with Copyright Clearance Center (CCC) to collect author charges. If fees apply to your paper, you will receive a message from no-reply@copyright.com with further instructions. For questions related to paying charges through RightsLink, please contact CCC at ASM_Support@copyright.com or toll free at +1-877-622-5543. CCC makes every attempt to respond to all emails within 24 hours.

ASM Membership: Corresponding authors may join or renew ASM membership to obtain discounts on publication fees. Need to upgrade your membership level? Please contact Customer Service at Service@asmusa.org.

Embargo Policy: A press release may be issued as soon as the manuscript is posted on the Spectrum Latest Articles webpage. The corresponding author will receive an email with the subject line "ASM Journals Author Services Notification" when the article is available online.

Cover Image Submissions: If you would like to submit a potential Cover Image, please email a file and a short legend to spectrum@asmusa.org. Please note that we can only consider images that (i) the authors created or own and (ii) have not been previously published. By submitting, you agree that the image can be used under the same terms as the published article. Image File requirements: TIF/EPS, 7.5 inches wide by 8.25 inches tall (at least 2,250 pixels wide by 2,475 pixels tall), minimum 300 dpi resolution (600 dpi preferred), RGB, and no figure elements, e.g., arrows or panel labels. The legend should be a short description of the image, 1-2 sentences recommended. Please use the ASM Journals Cover Image Sizing Tool (https://journals.asm.org/pb-assets/html/asm-cover-sizing-tool.html) to ensure that your proposed cover image meets our size requirements.

The ASM Journals program strives for constant improvement in our submission and publication process. Please tell us how we can improve your experience by taking this quick Author Survey.

Thank you for submitting your paper to Spectrum.

Sincerely,
Paul Luethy
Editor
Microbiology Spectrum
